# Cancer Pain Assessment and Management

**DOI:** 10.1038/sj.bjc.6601983

**Published:** 2004-07-27

**Authors:** B Jack, L Chapman

**Affiliations:** 1 Edge Hill College, Liverpool/Marie Curie Centre, Liverpool, UK; 2 Queenscourt Hospice, Southport, UK

The incidence of cancer is rapidly increasing in developed countries with 70% of patients diagnosed dying as a result of the disease. More than 80% of those who die of cancer will develop severe pain, yet a large number of studies document that cancer pain remains poorly assessed and managed in many patients. Bruera and Portenoy state that the purpose of the book is to provide a comprehensive clinically orientated and scholarly review of this complex and multidimensional problem that will lead to an increased understanding and contribute to an improvement in care.

Cancer Pain Assessment and Management is complied by internationally recognised authors who are current leaders in cancer pain research. Contributions from experts from all aspects of cancer pain management including psychology, psychiatry, anaesthetics, neurosciences and rehabilitation medicine lead to the comprehensiveness of the text.

The book is clearly presented with appropriate diagrams to compliment the text. It is logically structured and contains 28 chapters organised into eight key sections. Each chapter is supported by impressive and current reference lists, which are an invaluable resource for clinical practice, research and education.

The first section contains a detailed yet clear overview of the pathophysiology of pain, its prevalence and possible reasons for undertreatment. A section follows this on specific cancer pain syndromes and assessment of pain, which includes the importance of making a multidimensional assessment, taking into account psychological and spiritual distress. The authors then go on to discuss both the pharmacological and nonpharmacological management of pain, including psychological and rehabilitative interventions. In a minority of cancer patients, invasive pain management procedures need to be considered. The chapters on anaesthesiological procedures and neurosurgical techniques provide a comprehensive review of the options and their possible adverse effects.

The use of antineoplastic therapies for the management of cancer pain is addressed. These chapters discuss the clinical considerations in palliative radiotherapy and chemotherapy, in terms of likely response and treatment burden to the patient. They will be a valuable source of information for the palliative care physician considering oncological referral for pain control.

Among the discussion of difficult pain problems, there is a detailed chapter on cancer pain and depression. This discusses the availability of assessment tools for depression including their sensitivity and specificity, along with the management of depression including psychosocial and pharmacological interventions. Chapters on neuropathic pain, breakthrough pain and bone pain provide details of a wide range of therapeutic options.

The text also has a section on the management of cancer pain in specific populations, with chapters dedicated to paediatrics and the care of the elderly. Interestingly, there is a whole chapter on the management of cancer pain for chemically dependent patients, which is timely and necessary. This stresses the need for a multidisciplinary approach to these patients, and includes guidelines for developing a treatment plan.

The final section covers some of the broader topics that influence the management of cancer pain, incorporating ethical and legal considerations. This includes the concept of futility, advance directives, ‘do not resuscitate’ orders and euthanasia. Other end of life issues including withholding/withdrawing therapy, the use of artificial hydration and nutrition and ventilator withdrawal are also discussed. Additionally, there is a section on clinical trials in pain research that is a useful and practical reference for anyone wanting to undertake research on cancer pain. The final chapter focuses upon the patient's family in cancer pain management. This acknowledges the growing role of family caregivers in the management of cancer patients, in meeting both physical and psychosocial needs, and includes a list of useful resources for carers that are available on the Internet.

This comprehensive and practical text clearly meets Bruera and Portenoy's purpose and provides an excellent resource for the management and assessment of cancer and additionally noncancer pain. It will be invaluable for all clinicians, whether updating themselves on pharmacology or looking for help with a difficult clinical situation, and no doubt will become a classic text in the area of cancer pain.

